# Laryngotracheal separation surgery in a patient with severe Angelman syndrome involving a 19.3 Mb deletion on 15q11.2–q14

**DOI:** 10.1002/ccr3.6545

**Published:** 2022-11-06

**Authors:** Yohei Horikawa, Shuichi Yatsuga, Takashi Ohya, Yuki Okamatsu

**Affiliations:** ^1^ Iizuka Hospital Department of Pediatrics Iizuka Japan; ^2^ Fukuoka University Department of Pediatrics Fukuoka Japan

**Keywords:** angelman syndrome, aspiration pneumonia, bedridden, chromosome 15, laryngotracheal separation

## Abstract

A severe Angelman syndrome (AS) patient with a very large deletion (19.3 Mb) at 15q11.2‐q14 required laryngotracheal separation, which is not a common surgery in AS. Comparative genomic hybridization‐based microarrays can be useful to confirm deletion size and clinical severity.

## 1 INTRODUCTION

Angelman syndrome (AS) is a rare genetic neurodevelopmental disorder characterized by severe developmental delay, epilepsy, ataxic gait, and frequent laughter or smiling. The syndrome results from a lack of function of the imprinted ubiquitin protein ligase E3A (UBE3A) gene on chromosome 15q11.2–q13.^1^, ^2^ The prevalence of AS is approximately 1 per 20,000 births.^3^ Four different molecular defects may lead to the AS phenotype. Of these, recurrent de novo interstitial deletion of chromosomal region 15q11–q13 represents the most common genetic mechanism, found in about 75% of individuals with the condition.^2^ Uniparental disomy (UPD), imprinting defects (IC), and UBE3A gene mutations are additional genetic causes.^2^ The severity of AS has been reported to vary among these genotypes, with the most common also being the most severe. Most individuals with AS are able to walk; no previously described cases have been severe enough to bedridden.^4^, ^5^ Although the clinical course of AS has been reported for both children and adults, we found no examples in the literature where laryngotracheal separation was required. Even among the most profound forms of AS, reports of gastrostomy are few.^5^, ^6^, ^7^ Here, we report a bedridden case who suffered frequent episodes of aspiration pneumonia necessitating ventilator management, and who ultimately required laryngotracheal separation (LTS).

## 2 CASE REPORT

Our report concerns a male patient with profound AS who presents with severe motor and intellectual disabilities. At the time of intervention, the patient was 19‐year‐old with a height of 153 cm and a weight of 28.8 kg. His perinatal history was 40 weeks and 3 days in utero, vaginal delivery, and an Apgar score of 7/9. At 13 months old, AS was suspected due to abnormal limb movements, sleep disorder, and seizures. A diagnosis of AS due to 15q11.2–q13 deletion was confirmed by fluorescence in situ hybridization (FISH). At 1 year and 10 months old, he was hospitalized for convulsive status epilepticus, managed with two antiepileptic drugs. He had recurrent tonic seizures and was treated with three antiepileptic drugs at 6 years and 1 month old. He was hospitalized again at the age of 6 years and 9 months, there diagnosed as a non‐convulsive status epilepticus. His last hospitalization due to a seizure was at age 12, although his seizures remain poorly managed. His scoliosis worsened from age 13 and is now severe.

He is unable to sit and is bedridden. Despite being able to smile, he lacks significant vocalization and is currently unable to communicate nonverbally. He requires assistance in all aspects of daily life. He has experienced episodes of aspiration pneumonia every 6 months from 16 years old; an episode at 17 years old required ventilator management. From age 18, the frequency of aspiration pneumonia increased to more than once every 2 months. We first tried nonsurgical treatment, including a nasogastric tube and prohibition of oral intake, but were unable to prevent the onset of aspiration pneumonia. Reduced thoracic mobility due to severe scoliosis may contribute to repeated aspiration pneumonia. LTS is a well‐known ideal anti‐aspiration procedure for separating upper and lower respiratory tract to prevent aspiration pneumonia.^8^, ^9^ Since LTS is a drawback of sacrificing phonation. The patient was given informed consent for this procedure. We decided to perform LTS at 19‐year‐old. We considered performing a gastrostomy at the same time but, since almost the entire gastric body lies in the left thoracic cavity, we concluded that a successful gastrostomy would be anatomically difficult. After LTS, our patient was able to wean off tube‐feeding through an elemental diet tube and is now living at home with oral intake. One‐year post‐surgery, he has not been re‐hospitalized for aspiration pneumonia. Due to the extreme severity of his AS symptoms, microarray‐based comparative genomic hybridization (aCGH) was performed at age 19, revealing a very large deletion (19.3 Mb) at 15q11.2–q14. A 5–7 Mb deletion is common in AS.^2^ Methylation analysis showed abnormalities and is used to know the parental origin of a deletion.

## 3 DISCUSSION

Several studies have reported clinical differences in AS according to genotype. The genetic pattern of AS etiologies is as follows: deletion, approximately 75%; paternal UPD, 1%–2%; point mutation, 5%–10%; imprinting defect, 1%–3%; and unknown, 10%–15%.^2^ The deletion type has the highest frequency and is also more likely to show a history of severe medical conditions. Keute et al. in 2021^10^ summarized nine previous studies on differences in clinical symptoms by genotype and reported that problems of seizures (incidence and frequency), mental development, and motor development were all more severe in the deletion type. Several other reports also showed that gastrointestinal reflux disease, scoliosis, and use of nasogastric feeding tube were more frequent in the deletion type.^5^, ^11^ In a study of genetically confirmed AS individuals aged between 18 and 83 years, only 3% were unable to walk.^6^ A separate study showed that 98% of adults with AS were able to walk.^12^ Many of the non‐ambulatory cases reported in^5^, ^13^ were assumed to be of deletion type.

Compared with published descriptions of deletion type AS cases,^4^, ^5^, ^11^, ^13^, ^14^, ^15^, ^16^, ^17^, ^18^ the present case is considered particularly severe in motor development and internal complications. We identified only one report of gastrostomy in AS and no report of tracheostomy.^7^ The genotype of our patient is of the deletion type, which is regarded as the most profound form of AS but not usually severe enough to require gastrostomy or tracheostomy. However, the clinical course of this case deviated significantly from that of the typical deletion type, and LTS proved necessary to address intractable aspiration. Additionally, aCGH revealed a 19.3 Mb deletion: a magnitude which has not been previously reported.

Prader–Willi syndrome (PWS) is a rare genetic disorder, which, similar to AS, is related to defects on chromosome 15. PWS is associated with a range of complex physical and behavioral problems, usually including hyperphagia. Deletions causing AS and the related PWS generally share a common distal breakpoint (BP3) but differ in their proximal breakpoint (BP1 or BP2).^19^ Accordingly, these deletions can be divided into two main groups: Class I, which span BP1–BP3 (~6 Mb, ~16 genes and various noncoding regions deleted, accounting for about 40% of deletions); and Class II, spanning BP2–BP3 (~5 Mb, ~12 genes and various noncoding regions deleted, ~55% of deletions). Atypical deletions (i.e., Class III and Class IV, accounting for ~5%) may span chromosomal segments longer than Del1 or shorter than Del2.^10^, ^14^, ^19^, ^20^ In both AS and PWS cohorts, patients with Class I deletions are reported to have increased severity of neurodevelopment disorders relative to Class II deletions.^14^, ^17^, ^19^, ^21^, ^22^ Sahoo et al. in 2007^20^ summarized four AS cases with large deletions, including one of 10.68 Mb. All four cases had severe phenotypes, with scores below the average for Class I and Class II deletion children in five key aspects: cognitive abilities, motor skills, communication skills, self‐help skills, and socialization skills.” Large deletions are associated with severe phenotypes because of the involvement of several genes (e.g., *APBA2*, *TJP1*, *TRPM1*, and *CHRNA7*) that may influence phenotypic outcome. The deletion size seen in our patient is larger than any previously reported, and the phenotype is as severe, or more severe, than any previous reports. We consider the unusual deletion length to be a key cause of the atypically profound symptoms and necessity for LTS. The specific clinical manifestations in deletion 15q14 are unknown in this case.

More generally, it may be possible to predict the severity of an individual AS patient's condition by confirming the deletion size with aCGH. Early follow‐up with gastrostomy and tracheostomy in severe AS cases, where clinically indicated, may reduce later medical complications.

## AUTHOR CONTRIBUTIONS

YH chose the case, reviewed the published literature, and wrote the case report article. SY was the corresponding author, supervised article preparation and submitting article. TO was supervised clinical neurological situation and its interpretation. YO was supervised clinical respiratory situation and its interpretation. All the authors read and approved the final article.

## CONFLICT OF INTEREST

The authors declare no conflict of interest.

## CONSENT

Written informed consent was obtained from the patient and/or guardians regarding publishing the manuscript, and the report was approved by the Ethics Committee of Iizuka Hospital.

## Data Availability

Data sharing is not applicable to this article as no new data were created or analyzed in this study.
